# The acute phase management of spinal cord injury affecting polytrauma patients: the ASAP study

**DOI:** 10.1186/s13017-022-00422-2

**Published:** 2022-04-25

**Authors:** Edoardo Picetti, Corrado Iaccarino, Raul Coimbra, Fikri Abu-Zidan, Giovanni D. Tebala, Zsolt J. Balogh, Walter L. Biffl, Federico Coccolini, Deepak Gupta, Ronald V. Maier, Ingo Marzi, Chiara Robba, Massimo Sartelli, Franco Servadei, Philip F. Stahel, Fabio S. Taccone, Andreas W. Unterberg, Marta Velia Antonini, Joseph M. Galante, Luca Ansaloni, Andrew W. Kirkpatrick, Sandro Rizoli, Ari Leppaniemi, Osvaldo Chiara, Belinda De Simone, Mircea Chirica, Vishal G. Shelat, Gustavo P. Fraga, Marco Ceresoli, Luca Cattani, Francesco Minardi, Edward Tan, Imtiaz Wani, Massimo Petranca, Francesco Domenichelli, Yunfeng Cui, Laura Malchiodi, Emanuele Sani, Andrey Litvin, Andreas Hecker, Vito Montanaro, Solomon Gurmu Beka, Salomone Di Saverio, Sandra Rossi, Fausto Catena

**Affiliations:** 1 Department of Anesthesia and Intensive Care, Parma University Hospital, Via Gramsci 14, 43100 Parma, Italy; 2 Department of Biomedical, Metabolic and Neural Sciences, University of Modena and Reggio Emilia, Modena, Reggio Emilia, Italy; 3 Comparative Effectiveness and Clinical Outcomes Research Center, Riverside University Health System Medical Center, Moreno Valley, CA USA; 4 Department of Surgery, Loma Linda University School of Medicine, Loma Linda, CA USA; 5 Department of Surgery, College of Medicine and Health Sciences, UAE University, Al-Ain, United Arab Emirates; 6 Department of General Surgery, Oxford University Hospitals NHS Foundation Trust, Headley Way, Headington, Oxford, UK; 7 Department of Traumatology, John Hunter Hospital, Newcastle, NSW Australia; 8 Discipline of Surgery, School of Medicine and Public Health, University of Newcastle, Newcastle, NSW Australia; 9 Department of Trauma and Acute Care Surgery, Scripps Memorial Hospital, La Jolla, CA USA; 10 Department of Surgery, Pisa University Hospital, Pisa, Italy; 11 Department of Neurosurgery, All India Institute of Medical Sciences, New Delhi, India; 12 Department of Surgery, University of Washington, Seattle, WA USA; 13 Department of Trauma, Hand and Reconstructive Surgery, Hospital of the Johann Wolfgang Goethe-University Frankfurt Am Main, Frankfurt am Main, Germany; 14 Department of Anaesthesia and Intensive Care, Policlinico San Martino, IRCCS for Oncology and Neuroscience, Genova, Italy; 15 Dipartimento Di Scienze Chirurgiche Diagnostiche Integrate, University of Genova, Genova, Italy; 16 Department of General Surgery, Macerata Hospital, Macerata, Italy; 17 Humanitas University, Pieve Emanuele, Milan, Italy; 18 Humanitas Clinical and Research Center - IRCCS, Rozzano, Milan Italy; 19 College of Osteopathic Medicine, Rocky Vista University, Parker, CO USA; 20 The Medical Center of Aurora, Aurora, CO USA; 21 Department of Intensive Care, Erasme Hospital, Université Libre de Bruxelles (ULB), Brussels, Belgium; 22 Department of Neurosurgery, University of Heidelberg, Heidelberg, Germany; 23 ECMO Team, Bufalini Hospital, Cesena, Italy; 24 Department of Biomedical, Metabolic and Neural Sciences, University of Modena and Reggio Emilia, Modena, Reggio Emilia, Italy; 25 Division of Trauma and Acute Care Surgery, Department of Surgery, University of California Davis, Sacramento, CA USA; 26 Department of General Surgery, University Hospital of Pavia, Pavia, Italy; 27 General, Acute Care, Abdominal Wall Reconstruction, and Trauma Surgery, Foothills Medical Centre, Calgary, AB Canada; 28 Surgery Department, Section of Trauma Surgery, Hamad General Hospital (HGH), Doha, Qatar; 29 Abdominal Center, Helsinki University Hospital and University of Helsinki, Helsinki, Finland; 30 General Surgery and Trauma Team, ASST Niguarda Milano, University of Milano, Milan, Italy; 31 Department of General and Metabolic Surgery, Poissy and Saint-Germain-en-Laye Hospitals, Poissy, France; 32 Department of Digestive Surgery, Centre Hospitalier Universitaire Grenoble Alpes, La Tronche, France; 33 Department of General Surgery, Tan Tock Seng Hospital, Singapore, Singapore; 34 Surgery Department, Faculdade de Ciências Médicas (FCM), Unicamp Campinas, Campinas, SP Brazil; 35 General Surgery Department, School of Medicine and Surgery, Milano-Bicocca University, Monza, Italy; 36 Department of Surgery, Radboud University Medical Centre, Nijmegen, The Netherlands; 37 Department of Minimal Access and General Surgery, Government Gousia Hospital, Srinagar, Kashmir India; 38 Department of Surgery, Tianjin Nankai Hospital, Nankai Clinical School of Medicine, Tianjin Medical University, Tianjin, China; 39 Department of Surgical Disciplines, Immanuel Kant Baltic Federal University, Regional Clinical Hospital, Kaliningrad, Russia; 40 Department of General and Thoracic Surgery, University Hospital Giessen, Giessen, Germany; 41 Ethiopian Air Force Hospital, Bishoftu, Oromia Ethiopia; 42 Department of General Surgery, Ospedale Civile “Madonna del Soccorso”, San Benedetto del Tronto, AP Italy; 43 Department of General and Emergency Surgery, “M. Bufalini” Hospital, Cesena, Italy

**Keywords:** Polytrauma, Traumatic spinal cord injury, Management

## Abstract

**Background:**

Few data on the management of acute phase of traumatic spinal cord injury (tSCI) in patients suffering polytrauma are available. As the therapeutic choices in the first hours may have a deep impact on outcome of tSCI patients, we conducted an international survey investigating this topic.

**Methods:**

The survey was composed of 29 items. The main endpoints of the survey were to examine: (1) the hemodynamic and respiratory management, (2) the coagulation management, (3) the timing of magnetic resonance imaging (MRI) and spinal surgery, (4) the use of corticosteroid therapy, (5) the role of intraspinal pressure (ISP)/spinal cord perfusion pressure (SCPP) monitoring and (6) the utilization of therapeutic hypothermia.

**Results:**

There were 171 respondents from 139 centers worldwide. A target mean arterial pressure (MAP) target of 80–90 mmHg was chosen in almost half of the cases [*n* = 84 (49.1%)]. A temporary reduction in the target MAP, for the time strictly necessary to achieve bleeding control in polytrauma, was accepted by most respondents [*n* = 100 (58.5%)]. Sixty-one respondents (35.7%) considered acceptable a hemoglobin (Hb) level of 7 g/dl in tSCI polytraumatized patients. An arterial partial pressure of oxygen (PaO_2_) of 80–100 mmHg [*n* = 94 (55%)] and an arterial partial pressure of carbon dioxide (PaCO_2_) of 35–40 mmHg [*n* = 130 (76%)] were chosen in most cases. A little more than half of respondents considered safe a platelet (PLT) count > 100.000/mm^3^ [*n* = 99 (57.9%)] and prothrombin time (PT)/activated partial thromboplastin time (aPTT) < 1.5 times the normal control [*n* = 85 (49.7%)] in patients needing spinal surgery. MRI [*n* = 160 (93.6%)] and spinal surgery [*n* = 158 (92.4%)] should be performed after intracranial, hemodynamic, and respiratory stabilization by most respondents. Corticosteroids [*n* = 103 (60.2%)], ISP/SCPP monitoring [*n* = 148 (86.5%)], and therapeutic hypothermia [*n* = 137 (80%)] were not utilized by most respondents.

**Conclusions:**

Our survey has shown a great worldwide variability in clinical practices for acute phase management of tSCI patients with polytrauma. These findings can be helpful to define future research in order to optimize the care of patients suffering tSCI.

**Supplementary Information:**

The online version contains supplementary material available at 10.1186/s13017-022-00422-2.

## Background

Traumatic spinal cord injury (tSCI) is a devastating condition with a worldwide annual incidence ranging from near 10–80 cases for 1 million people [1, 2]. The most frequent causes of tSCI are falls from height and road traffic collisions, with an association of multisystem trauma up to 80% in the latter case [1, 3]. From a pathophysiological point of view, tSCI and traumatic brain injury (TBI) have some similarities [3, 4]. In tSCI, as in TBI, we observe primary and secondary injuries; the latter, in particular, can be further exacerbated by dangerous secondary insults (hypoxia and hypotension) with possible higher severity in unstable polytrauma patients [3, 4]. Unfortunately, little is known regarding the acute phase management of tSCI patients with multisystem trauma. As in TBI, the therapeutic choices in the first hours can have a deep impact on the outcome and prognosis of tSCI patients. For these reasons, we conducted an international survey investigating the practices in the acute phase management in polytrauma patients with associated SCI.

## Methods

### Ethical considerations

This survey addresses the acute phase management practices in polytrauma patients having SCI. Participants voluntarily agreed to join the survey. Therefore, this study did not need an ethical approval. Participants did not receive compensation for their participation in the survey; all those who agreed are identified as contributors at the end of the manuscript.

### Study design

This is a cross-sectional structured survey among the members of the World Society of Emergency Surgery (WSES) and the European Association of Neurological Surgeons (EANS).

### Sample size

This survey was distributed to the WSES and EANS members through their respective websites. Accordingly, sample size calculation was not needed and response rate could not be calculated as it used the media for communication.

### Questionnaire design

This online questionnaire had 29 questions (Additional file 1). It was divided into 7 sections which were: (1) demographic (questions 1–6), (2) hemodynamic and respiratory management (questions 7–13), (3) coagulation management (questions 14–16), (4) timing of magnetic resonance imaging (MRI) and surgical spinal decompression/stabilization (questions 17–21), (5) use of corticosteroid therapy (question 22), (6) the role of intraspinal pressure (ISP)/spinal cord perfusion pressure (SCPP) monitoring [with/without cerebrospinal fluid (CSF) drainage] (questions 23–27) and (7) utilization of therapeutic hypothermia (questions 28–29).

The questionnaire was written initially by two authors (EP and FC). An international panel of topic experts (number = 15) critically read and finalized the questionnaire. The final version of the survey was endorsed by the WSES.

### Distribution of the survey and data collection

An invitation to participate in the questionnaire was announced and distributed through a link in the WSES and the EANS websites during the period of November 1, 2020 through March 31, 2021. Furthermore, investigators targeted physicians who are involved in the acute care of polytrauma patients with tSCI [American Spinal Injury Association (ASIA) impairment scale grade A–D without TBI]. The online entered data were stored in a database which was only accessed by the principal investigators and was protected by a secure password.

### Statistical analysis

Data were downloaded from the online database, stored in an Excel file (Microsoft, Redmont, USA), and revised to assure the accuracy of the data. Only complete questionnaires were included in the final analysis. Descriptive statistics are reported as number (percentage). Comparisons between neurosurgeons versus non-neurosurgeons and between centers with an high admission trauma rate (> 250 polytrauma patients/year) versus low admission rate (< 250 polytrauma patients/year) were planned. Chi Square test or Fisher’s Exact test was used to compare categorical data of independent groups as appropriate. Cells with small values (0–3) were grouped with adjacent cells, where clinically reasonable. When grouping was not feasible, the cells were removed (grouped and removed cells are shown in the tables). Considering the exploratory and descriptive nature of the study, we did not find it necessary to correct for multiple comparisons, as it would be in the context of an experimental hypothesis testing that has been specified a priori [5]. In R × C tables, if the overall statistical test was significant, a post hoc test to detect the source of significance was done with the Fisher’s Exact test as suggested by Shan et al. [6], with the Hochberg’s [7] method to adjust for multiple comparisons. All analyses were performed using STATA 13.0 (STATA Corp, College Station, TX) software.

## Results

The number of respondents was 171 from 139 centers in 42 countries worldwide. The majority of respondents were from Italy [*n* = 35 (20.5%)], USA [*n* = 33 (19.3%)] and Qatar [*n* = 16 (9.4%)] (Additional file 2: Table S1). Baseline characteristics of the survey participants are shown in Table 1. The majority of respondents were neurosurgeons [*n* = 61 (35.7%)] and Emergency/Trauma surgeons [*n* = 57 (33.3%)]. One hundred and twelve respondents (65.5%) worked in a level I trauma center.

**Table 1:** Baseline characteristics of the survey participants

	Total
	n (%)
*Speciality*	
Int Care	25 (14.6)
Anesth	8 (4.7)
Em Med	5 (2.9)
E/T Surg	57 (33.3)
N Surg	61 (35.7)
Orth	10 (5.8)
other	5 (3)
*Years of practice with tSCI*	
< 5	26 (15.2)
6–10	37 (21.6)
11–15	38 (22.2)
16–20	18 (10.5)
21–25	22 (12.9)
> 25	30 (17.5)
*Trauma Center Level*	
I	112 (65.5)
II	21 (12.3)
III	38 (22.2)
*Major Trauma/year*	
< 50	17 (9.9)
50–100	35 (20.5)
100–250	38 (22.2)
250–500	34 (19.9)
> 500	47 (27.5)
*Pts with tSCI/year*	
< 20	42 (24.6)
20–30	43 (25.1)
30–40	31 (18.1)
40–50	22 (12.9)
> 50	33 (19.3)

*Int Care* intensive care, *Anesth* anesthesia, *Em Med* emergency medicine, *E/T surg* emergency trauma surgery, *N surg* neurosurgery, *Orth* orthopedics, *tSCI* traumatic spinal cord injury, *Pts* patients

### Cardiorespiratory management

Target mean arterial pressure (MAP) and hemoglobin (Hb) levels in polytrauma patients with tSCI are reported in Table 2. A target MAP of 80–90 mmHg was chosen in less than half of cases [*n* = 84 (49.1%)]. Sixty-eight respondents (39.8%) kept a default target MAP for at least 72 h. For the time strictly necessary to achieve bleeding control in polytrauma, a temporary reduction in the MAP target, was accepted by the majority of respondents [*n* = 100 (58.5%)]. Sixty-one respondents (35.7%) considered acceptable a Hb target of 7 g/dl in tSCI polytraumatized patients. The presence of tSCI in the setting of polytrauma did not change the Hb target [*n* = 125 (73.1%)]. The arterial partial pressure of oxygen (PaO_2_) and carbon dioxide (PaCO_2_) targets in polytrauma patients with tSCI are reported in Table 2. A PaO_2_ of 80–100 mmHg [*n* = 94 (55%)] and a PaCO_2_ of 35–40 mmHg [*n* = 130 (76%)] were chosen in most cases.

**Table 2:** Cardiorespiratory and coagulation management

	Total
	n (%)
*MAP target in polytrauma with tSCI*	
60–70 mm Hg	14 (8.2)
70–80 mm Hg	40 (23.4)
80–90 mm Hg	84 (49.1)
90–100 mm Hg	32 (18.7)
Other	1 (0.6)
*Time length of MAP target*	
24 h	18 (10.5)
48 h	26 (15.2)
72 h	68 (39.8)
4 d	5 (2.9)
5 d	17 (9.9)
6 d	1 (0.6)
7 d	34 (19.9)
Other	2 (1.2)
*Reduction in MAP target to achieve bleeding control*	
Yes	100 (58.5)
No	71 (41.5)
*Hb target in polytrauma without tSCI*	
7 g/dL	61 (35.7)
8 g/dL	47 (27.5)
9 g/dL	31 (18.1)
10 g/dL	31 (18.1)
Other	1 (0.6)
*Hb target in case of tSCI*	
Does not change	125 (73.1)
Increases	43 (25.1)
Decreases	3 (1.8)
*PaO*_*2*_* target*	
60–80 mm Hg	22 (12.9)
80–100 mm Hg	94 (55.0)
100–120 mm Hg	43 (25.1)
> 120 mm Hg	4 (2.3)
Other	8 (4.7)
*PaCO*_*2*_* target*	
< 35 mm Hg	14 (8.2)
35–40 mm Hg	130 (76.0)
40–45 mm Hg	19 (11.1)
> 45 mm Hg	0 (0.0)
other	8 (4.7)
*PLTs count target*	
> 50.000/μL	59 (34.5)
> 100.000/μL	99 (57.9)
> 250.000/μL	13 (7.6)
*PT/aPTT target for tSCI Surgery*	
< 1.2 normal control	81 (47.4)
< 1.5 normal control	85 (49.7)
< 1.8 normal control	5 (2.9)
*Usefulness of POC test*	
Yes	109 (63.7)
No	62 (36.3)

*MAP* mean arterial pressure, *tSCI* traumatic spinal cord injury, *Hb* hemoglobin, *PaO*_*2*_ arterial partial pressure of oxygen, *PaCO*_*2*_ arterial partial pressure of carbon dioxide, *PLTs* platelets, *PT* prothrombin time, *aPTT* activated partial thromboplastin time, *POC* point-of-care

### Coagulation management (Table 2***)***

Near half of respondents considered safe a platelet (PLT) count > 100.000/mm^3^ [*n* = 99 (57.9%)] and prothrombin time (PT)/activated partial thromboplastin time (aPTT) < 1.5 times the normal control [*n* = 85 (49.7%)] in tSCI polytrauma patients needing spinal surgery (decompression/stabilization). Point-of-care (POC) tests [i.e., thromboelastography (TEG) and rotational thromboelastometry (ROTEM)] were also considered useful in this scenario [*n* = 109 (63.7%)].

### MRI and spinal surgery (decompression/stabilization) timing (Table 3***)***

**Table 3:** MRI/spinal surgery timing, ISP/SPP monitoring and neuroprotective therapies

	Total
	n (%)
*Spinal surgery after intracranial, hemodynamic and respiratory stabilization?*	
Yes	158 (92.4)
No	13 (7.6)
*MRI after intracranial, hemodynamic and respiratory stabilization?*	
Yes	160 (93.6)
No	11 (6.4)
*Timing of MRI in ASIA grade A-D*	
Within 3 h	74 (43.3)
Within 6 h	38 (22.2)
Within 12 h	20 (11.7)
Within 24 h	20 (11.7)
Within 48 h	4 (2.3)
Within 72 h	6 (3.5)
Other	9 (5.3)
*Timing of spinal decompression/stabilization in ASIA grade A*	
Within 6 h	48 (28.1)
Within 12 h	26 (15.2)
Within 24 h	54 (31.6)
Within 48 h	19 (11.1)
Within 72 h	13 (7.6)
Other	11 (6.4)
*Timing of spinal decompression/stabilization in ASIA grade B-D*	
Within 6 h	58 (33.9)
Within 12 h	31 (18.1)
Within 24 h	57 (33.3)
Within 48 h	15 (8.8)
Within 72 h	8 (4.7)
Other	2 (1.2)
*Corticosteroids therapy in tSCI*	
Yes as NASCIS II/III	47 (27.5)
Yes but lower than NACSIS	18 (10.5)
No	103 (60.2)
Other	3 (1.8)
*Monitoring of ISP/SPP in tSCI*	
Frequently	8 (4.7)
In few cases	15 (8.8)
Never	148 (86.5)
*Is ISP/SPP monitoring useful in tSCI?*	
Yes	87 (50.9)
No	84 (49.1)
*CSF drainage in tSCI*	
Yes	35 (20.5)
No	136 (79.5)
*Therapeutic hypothermia in tSCI*	
Frequently	3 (1.8)
In few cases	31 (18.1)
Never	137 (80.1)
*Is therapeutic hypothermia useful in tSCI?*	
Yes	45 (26.3)
No	126 (73.7)

*MRI* magnetic resonance imaging, *ASIA* American Spinal Injury Association, *tSCI* traumatic spinal cord injury, *ISP* intraspinal pressure, *SPP* spinal perfusion pressure, *CSF* cerebrospinal fluid, *NASCIS* National Acute SCI study

MRI [*n* = 160 (93.6%)] and spinal surgery (decompression/stabilization) [*n* = 158 (92.4%)] should be performed after intracranial, hemodynamic and respiratory stabilization by the majority of respondents in tSCI polytraumatized patients. MRI could be performed within 3 h from the trauma [*n* = 74 (43.3%)]. The most frequent answers regarding timing for spinal surgery were within 24 h [*n* = 54 (31.6%)] and within 6 h [*n* = 48 (28.1%)] in ASIA grade A. Similarly, spinal surgery could be performed within 6 h [*n* = 58 (33.9%)] and within 24 h [*n* = 57 (33.3%)] in ASIA grade B–D.

### Corticosteroid therapy (Table 3)

Corticosteroids were not utilized by the majority of respondents [*n* = 103 (60.2%)]. When used, these were administered as in the National Acute Spinal Cord Injury Studies (NASCIS II and III) [8, 9] [*n* = 47 (27.5%)] or at a lower dose [*n* = 18 (10.5%)].

## ISP/SCPP monitoring (Table 3)

ISP/SCPP monitoring was generally not utilized [*n* = 148 (86.5%)] despite being considered useful by about half of the respondents [*n* = 87 (51%)].

The CSF drainage in tSCI was also utilized in few cases [*n* = 35 (20.5%)].

### Therapeutic hypothermia (Table 3)

Therapeutic hypothermia was never utilized in tSCI polytrauma patients in most cases [*n* = 137 (80%)] and considered not useful [*n* = 126 (73.7%)].

### Neurosurgeons versus non-neurosurgeons (Table 4)

**Table 4:**
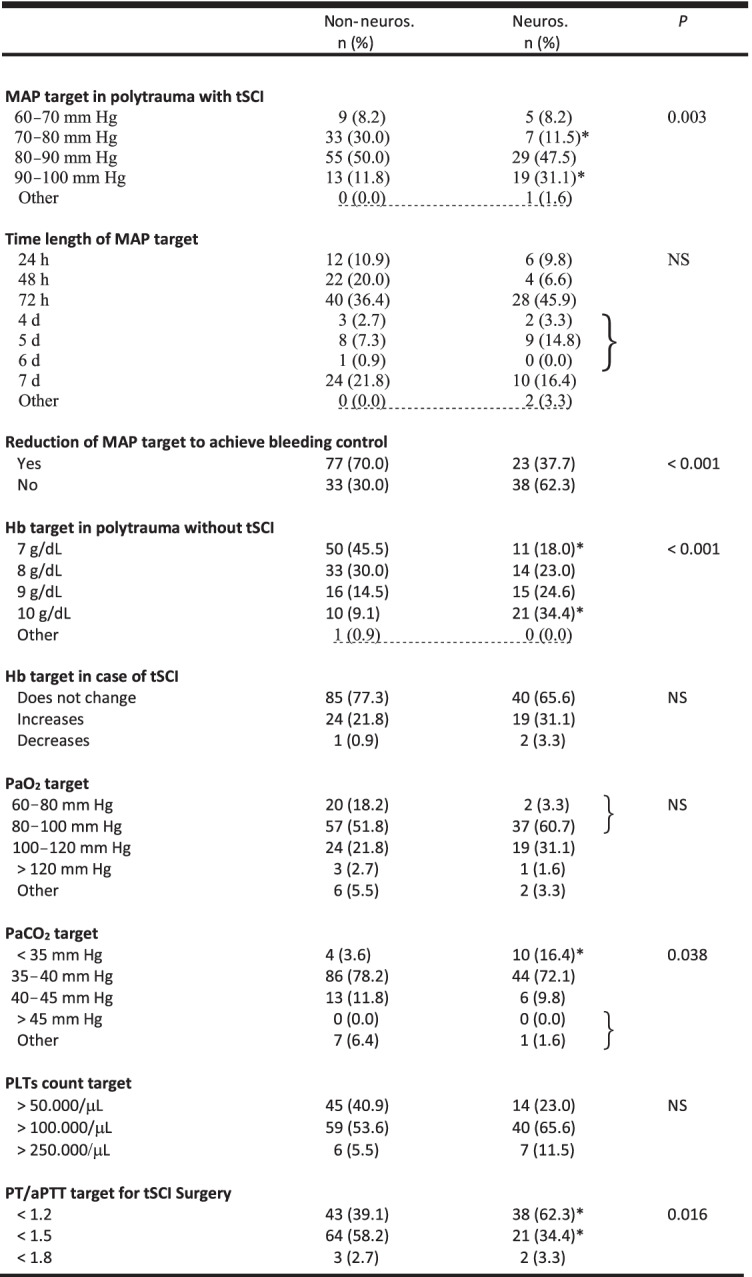

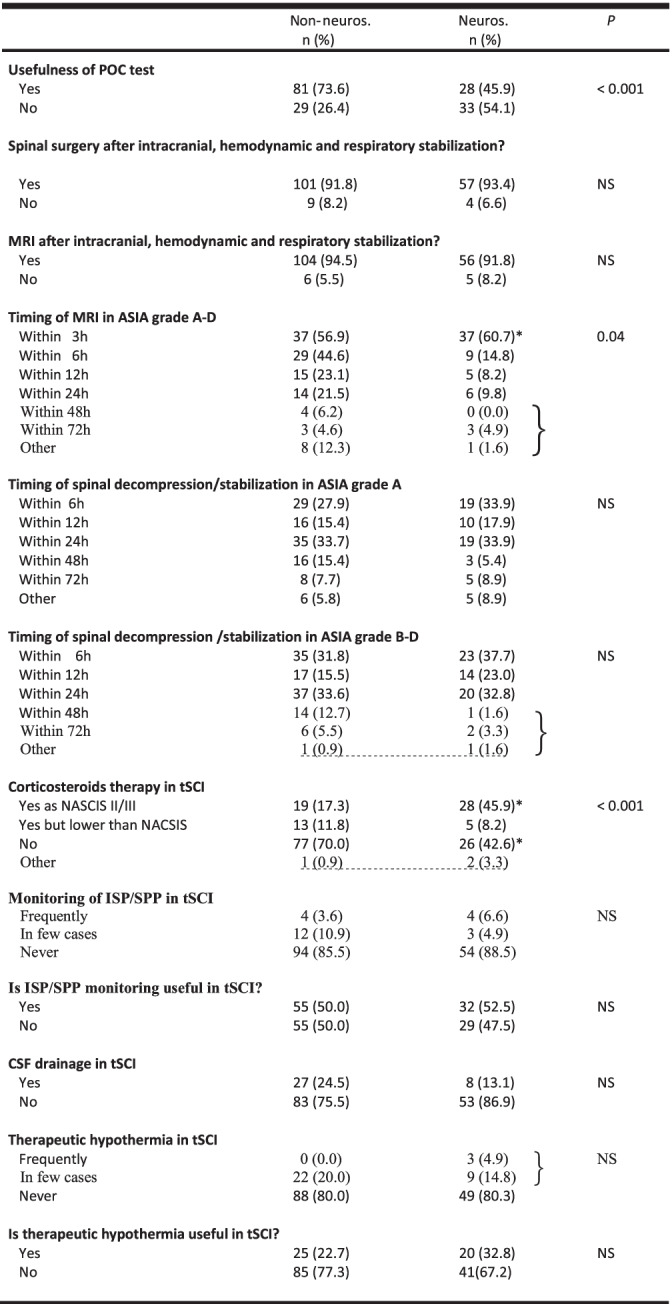
Comparison of neurosurgeons vs. non-neurosurgeons

*neuros* neurosurgeons, *MAP* mean arterial pressure, *tSCI* traumatic spinal cord injury, *Hb* hemoglobin, *PaO*_*2*_ arterial partial pressure of oxygen, *PaCO*_*2*_ arterial partial pressure of carbon dioxide, *PLTs* platelets, *PT* prothrombin time, *aPTT* activated partial thromboplastin time, *POC* point-of-care, *MRI* magnetic resonance imaging, *ASIA* American Spinal Injury Association, *ISP* intraspinal pressure, *SPP* spinal perfusion pressure, *CSF* cerebrospinal fluid, *NASCIS* National Acute SCI study, *NS* not significant

* = *P* < 0.05 vs Non-Neuros at the post hoc analysis

A curly bracket indicates that the cells were grouped for statistical purposes

Dotted underline means that cells were removed for statistical purposes

Considering the comparison between neurosurgeons and non-neurosurgeons, the statistically significant differences refer to:

- Target MAP (more non-neurosurgeons considered safe a target MAP of 70–80 mmHg and more neurosurgeons considered safe a target MAP of 90–100 mmHg)
- Temporary reduction in the target MAP to achieve bleeding control (more in the non-neurosurgeons group)
- Target Hb (more non-neurosurgeons considered safe a target Hb of 7 g/dl and more neurosurgeons considered safe a target Hb of 10 g/dl)
- Target PaCO_2_ (more neurosurgeons considered safe a target PaCO_2_ < 35 mmHg)
- Target PT/aPTT (more neurosurgeons considered safe a target PT/aPTT < 1.2 normal control and more non-neurosurgeons considered safe a target PT/aPTT target < 1.5 normal control)
- POC tests (more useful in the non-neurosurgeons group)
- Timing of MRI in stable tSCI polytrauma patients (ASIA grade A–D) (more neurosurgeons suggested performing MRI within 3 h after injury)
- Corticosteroid therapy (more non-neurosurgeons did not utilize corticosteroid therapy, and more neurosurgeons utilized corticosteroids as in NASCIS II /III trials).

### Trauma centers with polytrauma patients’ admission < 250/year versus > 250/year (Table 5***)***

**Table 5:**
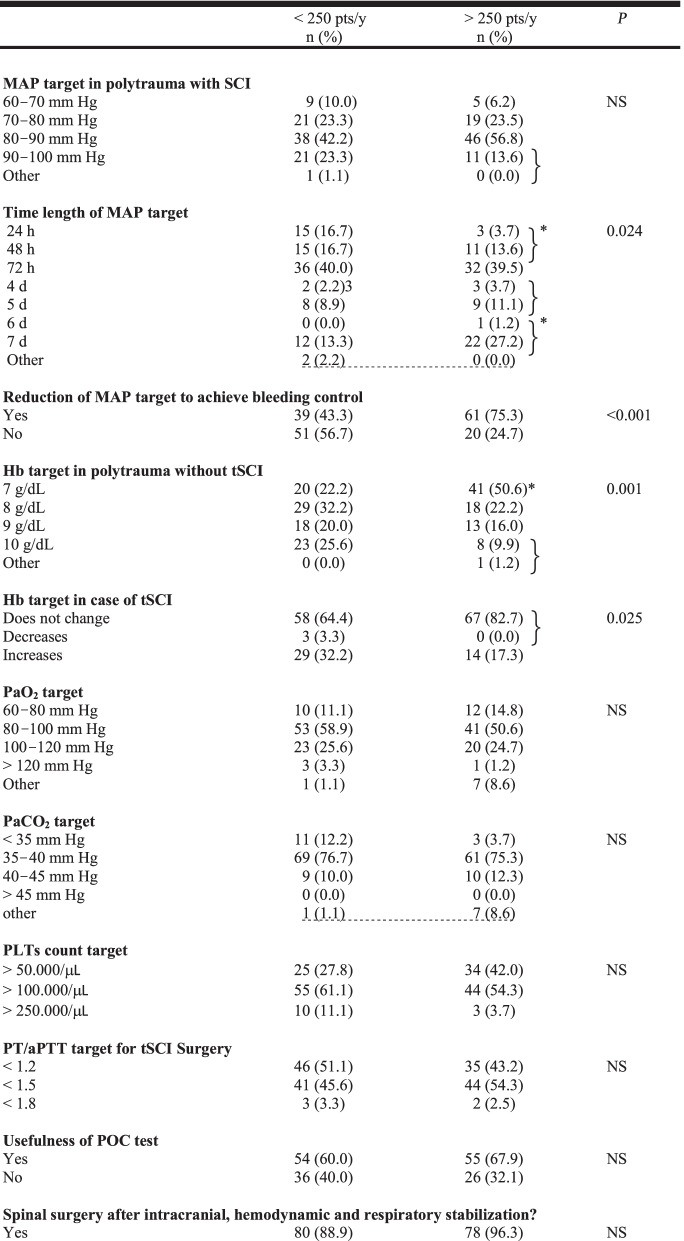

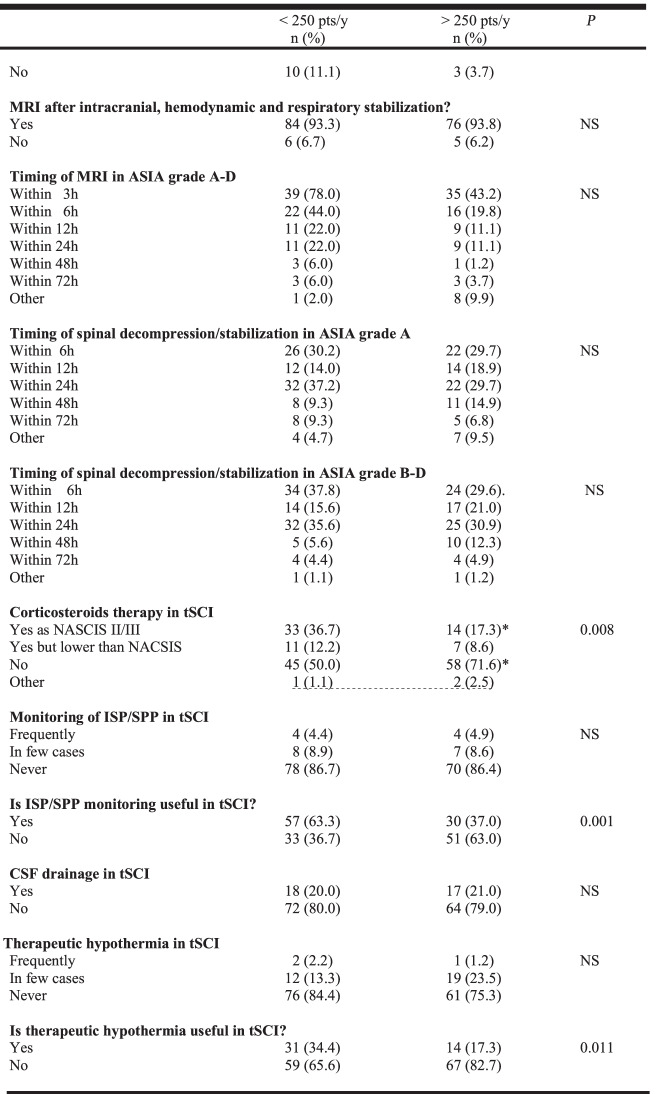
Comparison of trauma centers with polytrauma patients admission < 250/year versus > 250/year

*pts* patients, *y* year, *MAP* mean arterial pressure, *tSCI* traumatic spinal cord injury, *Hb* hemoglobin, *PaO*_*2*_ arterial partial pressure of oxygen, *PaCO*_*2*_ arterial partial pressure of carbon dioxide, *PLTs* platelets, *PT* prothrombin time, *aPTT* activated partial thromboplastin time, *POC* point-of-care, *MRI* magnetic resonance imaging, *ASIA* American Spinal Injury Association, *ISP* intraspinal pressure, *SPP* spinal perfusion pressure, *CSF* cerebrospinal fluid, *NASCIS* National Acute SCI study, *NS* not significant

* = *P* < 0.05 versus < 250 pts/y at the post hoc analysis

A curly bracket indicates that the cells were grouped for statistical purposes

Dotted underline means that cells were removed for statistical purposes

Regarding the comparison between trauma centers with polytrauma patients’ admission < 250/year and > 250/year, the statistically significant differences refer to:

- Maintenance of target MAP (more respondents in the group with < 250 pts/year maintained target MAP for 24/48 h and fewer respondents in the group with < 250 pts/years maintained the target MAP for more than 6 days)
- Temporary reduction in the target MAP to achieve bleeding control (more in the > 250/year group)
- Target Hb (more physicians working in the > 250/year group considered safe a target Hb of 7 g/dl in polytrauma patients without tSCI; the presence of tSCI led to an increase in the target Hb in the < 250/year group)
- Corticosteroids therapy (more physicians in the > 250/year group did not utilize corticosteroids therapy, and more physicians in the < 250/year group utilized corticosteroids as in the NASCIS II/III studies)
- ISP/SCPP monitoring (more useful in the < 250/year group)
- Therapeutic hypothermia (less useful in the > 250/year group)

## Discussion

This international survey provides important information regarding worldwide acute phase management practices in polytrauma tSCI patients with particular focus on (1) cardiorespiratory management, (2) coagulation management, (3) MRI/spinal surgery timing, (4) corticosteroid therapy, (5) ISP/SCPP monitoring and (6) therapeutic hypothermia.

### Cardiorespiratory management

A cardiorespiratory dysfunction (arterial hypotension, hypoxia, etc.) is frequently observed after tSCI, particularly when the injury occurs at high spinal cord levels, and is associated with an unfavourable neurological outcome [1]. This condition can be exacerbated further in unstable polytrauma patients [3]. The most recent guidelines by the Congress of Neurological Surgeons (CNS) for the management of tSCI patients recommend the maintenance of MAP between 85 and 90 mm Hg for the first 7 days following an acute cervical SCI (Level III) [10]. This recommendation is poorly adopted by most of our respondents which consider safe the maintenance of a MAP value of 80–90 mmHg only for 3 days. Higher than recommended MAP values are deemed safe for neurosurgeons, maybe reflecting a greater attention for spine perfusion. These data suggest that additional educational efforts are required to increase clinical awareness concerning established and published recommendations to improve outcomes in tSCI patients.

Traditionally, the “golden hour” treatment of injured patients with or at risk of hemorrhagic shock consisted in an aggressive fluid resuscitation, at a 3:1 ratio with the estimated blood loss, to maintain a normal MAP to allow peripheral tissue perfusion. While this represented a huge step forward to decrease mortality from trauma, soon it was demonstrated that massive volume replacement has its drawbacks in terms of tissue oedema and impaired metabolic response; therefore it was speculated that aggressive resuscitation would jeopardize our efforts to rescue hemorrhagic patients. Permissive hypotension was introduced with the aim to reduce the risks of fluid overload while maintaining an adequate tissue oxygenation. However, the optimal tissue perfusion pressure has not been determined yet [11]. While it has been suggested to maintain a MAP around 70 mmHg in torso trauma patients, this target has been considered insufficient to maintain brain perfusion in patients with severe head trauma [12]. In the literature there is no specific evidence to guide the application of permissive hypotension to spine trauma but considering the frequent association between spine and head trauma, it seems logical to make any effort to maintain a MAP around 85–90 mmHg.

For the time strictly necessary to achieve bleeding control in polytrauma, a temporary reduction in the target MAP, was accepted by little more than half of respondents and more non-neurosurgeons and physicians working in high- volume centers. Probably, the choice of the respondents could be influenced by the increase in worldwide utilization of damage control resuscitation (DCR) protocols in polytrauma patients [13]. However, targeted parameters for maintenance of blood pressure should be higher in polytrauma patients with tSCI.

Guidelines for the management of tSCI patients do not refer to optimal Hb values, and data from high-quality studies in this setting are lacking [10, 14]. However, most respondents consider acceptable a target Hb of 7 g/dl in tSCI polytraumatized patients, and the presence of tSCI in the setting of polytrauma does not influence this strategy. This approach, mainly adopted by non-neurosurgeons and physicians working in high-volume centers, could reflect recommendations derived from different trauma guidelines [15, 16].

As for Hb values, data regarding optimal PaO_2_ and PaCO_2_ targets in tSCI polytrauma patients are lacking. In most cases, a PaO_2_ of 80–100 mmHg and a PaCO_2_ of 35–40 mmHg were chosen. This choice could be affected by what is recommended in patients with acute brain damage [17].

### Coagulation management

The most recent European guideline concerning the management of major hemorrhage and coagulopathy following trauma [16] recommended that PT and aPTT be maintained < 1.5 times the normal control (grade 1C) and the PLT count be maintained above 50,000/mm^3^ (grade 1C). In addition, the maintenance of a PLT count > 100,000/mm^3^ was also recommended for patients with ongoing bleeding and/or TBI (grade 2C) [14] and in the case of neurosurgery [18]. To our knowledge, no specific guidelines regarding coagulation management in tSCI patients have been published, to date. However, POC tests (i.e., TEG, ROTEM, etc.) are increasingly used to evaluate coagulation function in trauma patients with hemorrhagic complications [16, 19]. In particular, these tests can be utilized to obtain a rapid assessment of hemostasis, to assist in clinical decision-making and to provide critical information about specific coagulation deficiencies, especially in patients taking novel oral anticoagulants (NOACs) and in the evaluation of PLTs dysfunction induced by trauma and/or drugs [14, 19]. Most of the respondents are in accordance with these recommendations. Moreover, regarding PT and aPTT, predominantly neurosurgeons also have a more conservative approach.

### MRI/Spinal surgery timing

MRI, providing a detailed image of the spinal cord and related soft tissues, is very important in influencing the treatment and prognosis of tSCI patients [2, 20]. However, considering its duration of execution and technical characteristics, it may be dangerous in cardiorespiratory unstable polytrauma patients. For this reason, as also remarked by the majority of the respondents, it could be performed after intracranial, hemodynamic, and respiratory stabilization.

Recent guidelines suggest that MRI should be performed in adult patients with acute SCI: (a) before surgical intervention, when feasible, to facilitate improved clinical decision making (Quality of Evidence: Very Low, Strength of Recommendation: Weak) and (b) in the acute period following SCI, before or after surgical intervention, to improve prediction of neurologic outcome (Quality of Evidence: Low Strength of Recommendation: Weak) [20]. However, an accurate and precise timing for MRI in tSCI patients is not clearly defined and probably needs to be determined. For most of the respondents, particularly neurosurgeons, MRI could be performed within 3 h from the trauma in stable patients.

Recent studies suggest as early decompressive surgery (performed within 24 h from trauma) is associated with better neurological outcomes, thus highlighting the concept of “time is spine” [21, 22]. A more rapid approach (within 12 h or less) was also proposed in case of the incomplete spinal lesion (ASIA B–D) [23–25]. Recent guidelines “suggest that early surgery (< 24 h after injury) be considered as a treatment option in adult patients with traumatic central cord syndrome (Quality of Evidence: Low. Strength of Recommendation: Weak) and that early surgery be offered as an option for adult acute SCI patients regardless of level (Quality of Evidence: Low. Strength of Recommendation: Weak)” [26]. The majority of the respondents are in agreement with the timing as mentioned above, and a more rapid approach (< 6 h from trauma) was also preferred in cases of incomplete spinal lesions (ASIA B–D). The optimal timing of spinal surgery in tSCI polytrauma patients needs to be established and individualized after intracranial, hemodynamic and respiratory stabilization, as most of the respondents remarked.

### Corticosteroid therapy

The utilization of methylprednisolone sodium succinate (MPSS) after tSCI is a debated and controversial topic. Guidelines from the CNS [27] do not recommend its use at all (Level I), whereas guidelines from the AO spine [28] suggest: (1) “not offering a 24-h infusion of high-dose MPSS to adult patients who present after 8 h with acute SCI”; (2) “a 24-h infusion of high-dose MPSS to adult patients within 8 h of acute SCI as a treatment option,” and (3) “not offering a 48-h infusion of high-dose MPSS to adult patients with acute SCI.” The majority of the respondents agreed with the CNS guidelines. However, more neurosurgeons (compared with non-neurosurgeons) and more physicians working in low- volume centers utilize corticosteroids. This may reflect the contrast between the two guidelines [27, 28]. Probably this topic will have to be evaluated in future well-performed studies.

### ISP/SCPP monitoring

Recently, interest in ISP/SCPP monitoring was increased [29]. The ISP can be evaluated by surgically implanting an intradural extramedullary probe at the injury site [29–32]. In this way, it is possible to obtain SCPP (MAP-ISP) that can be considered a more accurate way to monitor spinal cord perfusion with respect to MAP, such as cerebral perfusion pressure (CPP) in TBI [29]. A SCPP > 50 mm Hg is proven to be a strong predictor of improved neurologic recovery following SCI [30, 32]. In this regard, SCPP could provide useful information to guide the hemodynamic management of acute SCI patients.

More data are also necessary to increase the use of this type of monitoring in daily clinical practice. The responses collected in the survey are consistent with this aspect. However, our results also reflect the paucity of data regarding the role of CSF drainage in acute SCI [33].

### Therapeutic hypothermia

Hypothermia, through various mechanisms, can play a role in preventing secondary injury after SCI [34]. Moreover, more data are necessary for its application in daily clinical practice [34]. Most of the respondents do not utilize this type of therapeutic approach or consider it useful.

### Limitations

We have to acknowledge that our study has several limitations *First,* the number of the respondents was relatively small. This may reflect a selection bias with those more interested in this area which limits its generalizability. *Second,* this survey reflects personal opinions and practices which may be subjective or affected by recall bias. *Third,* 60% of the responders were from three countries which represents a geographical bias. *Forth,* using a web-based survey with secondary distribution hinders the ability to calculate the response rate. However, we were encouraged to find that we obtained responses from 139 centers worldwide. *Finally*, to be more focused and to improve the response rate by making the questionnaire short we have defined specific important topics excluding other questions which may be equally important.

## Conclusions

Great worldwide great variability in clinical practices for acute phase management of tSCI patients with polytrauma was identified from the survey results. This finding can be helpful be helpful to optimize the care of patients having tSCI and to define future research questions to be answered.

## Supplementary Information

**Additional file 1.** Questionnaire.

**Additional file 2.** **Table S1** – Countries of respondents.

## Data Availability

The datasets used and/or analyzed during the current study are available from the corresponding author on reasonable request.

## Abbreviations

tSCI: Traumatic spinal cord injury
TBI: Traumatic brain injury
WSES: World Society of Emergency Surgery
EANS: European Association of Neurological Surgeons
MRI: Magnetic resonance imaging
ISP: Intraspinal pressure
SCPP: Spinal cord perfusion pressure
CSF: Cerebrospinal fluid
ASIA: American Spinal Injury Association
MAP: Mean arterial pressure
Hb: Hemoglobin
PaO_2_: Arterial partial pressure of oxygen
PaCO_2_: Arterial partial pressure of carbon dioxide
PLT: Platelet
PT: Prothrombin time
aPTT: Activated partial thromboplastin time
POC: Point-of-care
TEG: Thromboelastography
ROTEM: Rotational thromboelastometry
NASCIS: National Acute Spinal Cord Injury Study
DCR: Damage control resuscitation
NOACs: Novel oral anticoagulants
MPSS: Methylprednisolone sodium succinate
CNS: Congress of Neurological Surgeons
CPP: Cerebral perfusion pressure
