# Supplementary material for: The acute phase management of spinal cord injury affecting polytrauma patients: the ASAP study
Source: World J Emerg Surg. 2022 Apr 25;17:20. doi: 10.1186/s13017-022-00422-2 (PMC9036814; doi:10.1186/s13017-022-00422-2)
Supplement: Supplementary file 1 — Additional file 1. Questionnaire. [file 13017_2022_422_MOESM1_ESM.docx]

QUESTIONNAIRE

**1.** In which country do you practice?

**2.** Your primary specialty is:

- Intensive Care

- Anesthesia

- Emergency Medicine

- Emergency Surgery

- Neurosurgery

- Orthopedics

- other

**3.** Numbers of years of practice in the care of polytrauma patients with traumatic spinal cord injury (tSCI):

- < 5

- 6-10

- 11-15

- 16-20

- 21-25

- > 25

**4.** Your hospital is a Trauma center level:

- I

- II

- III

**5.** The annual number of major trauma admissions [Injury Severity Score (ISS) > 15] at your center is:

- < 50

- 50 - 100

- 100 - 250

- 250 - 500

- > 500

**6.** The annual number of major trauma patient admissions with associate tSCI (ASIA grade A-D) is:

- < 20

- 20 - 30

- 30 - 40

- 40 - 50

- > 50

***HEMODYNAMIC/RESPIRATORY MANAGEMENT***

**7.** In general, in polytrauma patients with tSCI (no TBI and ASIA grade A-D) you try to maintain a mean arterial pressure (MAP) of:

- 60-70 mmHg

- 70-80 mmHg

- 80-90mmHg

- 90-100 mmHg

- other (please specify)

**8.** How long after injury do you try to maintain the MAP target chosen in the previous question?

- 24 hours

- 48 hours

- 72 hours

- 4 days

- 5 days

- 6 days

- 7 days

- other (please specify)

**9a.** In unstable hemorrhagic polytrauma patients with tSCI (no TBI and ASIA grade A-D) do you consider reasonable to reduce the MAP target for the time necessary to achieve bleeding control?

- yes

- no

**9b.** If the answer to the previous question is YES, please indicate the new MAP target:

**10.** In general, in polytrauma patients with tSCI (no TBI and ASIA grade A-D) you try to maintain a hemoglobin (Hb) level of:

- > 7 g/dl

- > 8 g/dl

- > 9 g/dl

- > 10 g/dl

- other (please specify)

**11.** Considering the previous question, the presence of tSCI in polytrauma patients:

- does not change the Hb target when compared to polytrauma without

tSCI

- increase the Hb target when compared to polytrauma without tSCI

- decrease the Hb target when compared to polytrauma without tSCI

**12.** In general, in polytrauma patients with tSCI (no TBI and ASIA grade A-D) you try to maintain an arterial partial pressure of oxygen (PaO_2_) of:

- 60-80 mmHg

- 80-100 mmHg

- 100-120 mmHg

- > 120 mmHg

- other (please specify)

**13.** In general, in polytrauma patients with tSCI (no TBI and ASIA grade A-D) you try to maintain an arterial partial pressure of carbon dioxide (PaCO_2_) of:

- < 35 mmHg

- 35-40 mmHg

- 40-45 mmHg

- > 45 mmHg

- other (please specify)

***COAGULATION MANAGEMENT (SURGICAL SPINAL DECOMPRESSION/STABILIZATION)***

**14.** In our daily clinical practice, in polytrauma patients with tSCI (no TBI and ASIA grade A-D) needing surgical spinal decompression/stabilization you consider safe a platelet count of:

- > 50.000 / mm^3^

- > 100.000 / mm^3^

- > 150.000 / mm^3^

**15.** In our daily clinical practice, in polytrauma patients with tSCI (no TBI and ASIA grade A-D) needing surgical spinal decompression/stabilization you consider safe a Prothrombin Time (PT)/ Activated Partial Thromboplastin Time (APTT) of:

- < 1,2 times the normal control
- < 1,5 times the normal control
- < 1,8 times the normal control

**16.** In polytrauma patients with tSCI (no TBIand ASIA grade A-D) needing surgical spinal decompression/stabilization (without active bleeding) do you consider useful the utilization of Point-of-Care tests (ie. TEG, ROTEM) to optimize the coagulation status?

- yes

- no

***TIMING OF MRI AND SURGICAL SPINAL DECOMPRESSION/STABILIZATION***

**17.** In general, in polytrauma patients with tSCI (ASIA grade A-D) spinal surgery should be performed after intracranial, hemodynamic and respiratory stabilization. Do you agree?

- yes

- no

**18 -** In general, in polytrauma patients with tSCI (ASIA grade A-D) MRI should be performed after intracranial, hemodynamic and respiratory stabilization. Do you agree?

- yes

- no

**19.** In respiratory and hemodynamically stable polytrauma (no TBI) patients with tSCI (ASIA grade A) surgical spinal decompression/stabilization need to be performed:

- within 6 hours after injury

- within 12 hours after injury

- within 24 hours after injury

- within 48 hours after injury

- within 72 hours after injury

- others (please specify)

**20.** In respiratory and hemodynamically stable polytrauma (no TBI) patients with tSCI (ASIA grade B, C and D) surgical spinal decompression/stabilization need to be performed:

- within 6 hours after injury

- within 12 hours after injury

- within 24 hours after injury

- within 48 hours after injury

- within 72 hours after injury

- others (please specify)

**21.** In respiratory and hemodynamically stable polytrauma (no TBI) patients with tSCI (ASIA grade A-D) MRI need to be performed:

- within 3 hours after injury

- within 6 hours after injury

- within 12 hours after injury

- within 24 hours after injury

- others (please specify)

***CORTICOSTEROIDS THERAPY***

**22.** In polytrauma patients with tSCI (ASIA grade A-D) do you utilize corticosteroid therapy?

- yes as in NASCIS II/III studies

- yes but a lower doses respect to NASCIS II/III studies

- no

- other (please specify)

***INTRASPINAL PRESSURE/SPINAL CORD PERFUSION PRESSURE MONITORING***

**23.**  Do you monitor intraspinal pressure/spinal cord perfusion pressure in polytrauma patients with tSCI (ASIA grade A-D) at your center?

- yes, frequently

- yes, in few cases

- never

**24.** Do you consider useful the possibility to perform intraspinal pressure/spinal cord perfusion pressure monitoring in polytrauma patients with tSCI (ASIA grade A-D) ?

- yes

- no

**25.** In case of utilization of intraspinal pressure monitoring, you try to maintain an intraspinal pressure (mmHg) lower than:

**26.** In case of utilization of spinal cord perfusion pressure monitoring, you try to maintain a spinal perfusion pressure (mmHg) greater than:

**27.** Do you utilize spinal CSF drainage to control intraspinal pressure?

***THERAPEUTIC HYPOTHERMIA***

**28.** Do you utilize therapeutic hypothermia in hemodynamically stable polytrauma patients with tSCI (no TBI and ASIA grade A-D) at your center?

- yes, frequently

- yes, in few cases

- never

**29.** Do you consider useful the utilization of therapeutic hypothermia in hemodynamically stable polytrauma patients with tSCI (no TBI and ASIA grade A-D) ?

- yes

- no
