# Supplementary material for: The acute phase management of spinal cord injury affecting polytrauma patients: the ASAP study
Source: World J Emerg Surg. 2022 Apr 25;17:20. doi: 10.1186/s13017-022-00422-2 (PMC9036814; doi:10.1186/s13017-022-00422-2)
Supplement: Supplementary file 2 — Additional file 2. Table S1 – Countries of respondents. [file 13017_2022_422_MOESM2_ESM.docx]

**Table S1 –** Countries of respondents.

Abbreviations: UK =United Kingdom, U.S.A. = United States of America.
